# Postpartum indicators of stress injury and inflammatory factors in high-risk pregnancies by oxytocin combined with cervical balloon dilators

**DOI:** 10.1016/j.clinsp.2025.100668

**Published:** 2025-07-10

**Authors:** Ruihong Lan, Mengdi Xue, Yihong Yu, Xiaoqing Huang, Humin Gong

**Affiliations:** a Department of Obstetrics, Hainan General Hospital, Hainan Affiliated Hospital of Hainan Medical University, Haikou, Hainan, China; b Hainan Medical University, Haikou, Hainan, China

**Keywords:** High-risk pregnancy, Oxytocin, Cervical balloon dilators, Inflammatory response, Stress response

## Abstract

**Objective:**

This work analyzes the effect of the combination of Oxytocin (OXT) combined with cervical balloon dilators on stress injury indicators and inflammatory factors in pregnant women with high-risk pregnancies.

**Methods:**

Ninety-three pregnant women with high-risk pregnancies from June 2021 to November 2023 were collected and analyzed retrospectively, of which 43 cases were induced by OXT (control group), and 50 cases were induced by OXT combined with cervical balloon dilators (observation group). The differences in blood pressure and Heart Rate (HR) between the two groups were compared, and the pre-and post-treatment oxidative stress indicators Norepinephrine (NE), Adrenaline (AD), and Cortisol (Cor) and the inflammatory factors Tumor Necrosis Factor-α (TNF-α), Interleukin-1β (IL-1β), Interleukin-6 (IL-6), and Prostaglandin E2 (PGE2). In addition, postpartum complications were counted in both groups.

**Results:**

The observation group had higher BP and lower HR than the control group (*p* < 0.05). After treatment, NE, AD, Cor, TNF-α, IL-1β, IL-6, and PGE2 increased in both groups, but the observation group was lower than the control group (*p* < 0.05). In addition, there was no difference in the incidence of postpartum complications between the observation group and the control group (*p* > 0.05).

**Conclusion:**

OXT combined with cervical balloon dilators can effectively ameliorate oxidative stress injury and reduce inflammatory response in high-risk pregnancy women.

## Highlights

• Oxytocin combined with cervical balloon dilators improves postpartum stress in high-risk pregnancies.
• Oxytocin in combination with cervical balloon dilators improves postpartum inflammation in high-risk pregnancies.
• Oxytocin in combination with cervical balloon dilators may improve hemodynamics in high-risk pregnancies.
• Oxytocin combined with Cervical balloon dilators can shorten the duration of labor and improve the safety of delivery in high-risk pregnancies.

## Introduction

Influenced by the rapid development of society, changes in people's living habits, and adverse environmental factors, high-risk pregnancies have pregnancies increasingly common in clinical practice.^1^ Clinical statistics suggest that high-risk pregnancies now account for approximately 5 to 12 percent of all pregnancies, with higher rates in some developed countries.^2^ Due to the presence of various underlying diseases, the maternal and infant health of high-risk pregnant women is greatly threatened.^3^ Induction of labor, one of the commonly used clinical means to deal with high-risk pregnancies at present, terminates pregnancy to improve maternal and infant safety with drugs, instruments, and other stimulation methods, which is also of great significance for the reproductive health of postpartum women.^4^ Among them, cervical balloon dilators are commonly used midwifery devices in obstetrics, which simulate the compression of the cervix by the fetal head through mechanical stimulation, playing a softening and dilation role; moreover, through the physical dilation effect, the woman's cervix is stimulated, which can naturally and gradually expand or soften the cervix, improve the success rate of induced abortion, and reduce harm to the women.^5^ Oxytocin (OXT) is also a type of medication that promotes childbirth and has the effect of promoting regular uterine contractions.^6^ At present, studies have confirmed that the combination of the two can accelerate the delivery of high-risk pregnant women to a certain extent and increase the probability of successful induction of labor.^7^^,^^8^ However, its effects on postpartum stress injury are rarely reported.

As the authors all know, childbirth is a positive feedback regulatory behavior of the human body, accompanied by obvious neuropathic pain, which can cause severe stress reactions in the mother's body and activate a large number of inflammatory mediators, increasing the occurrence of various postpartum complications, such as postpartum intrauterine infection, postpartum hemorrhage, postpartum depression, etc.^9^ For high-risk pregnant women, the management of their postpartum stress injury and inflammatory reaction determines their rehabilitation quality.^10^

Therefore, while exploring the delivery safety of OXT combined with cervical balloon dilators for high-risk pregnant women, this study further analyzes the effect of this regime on postpartum stress injury and inflammatory reactions in mothers, providing effective guidance for future clinical applications.

## Materials and methods

### Design of study

The authors designed a retrospective analysis. The potential study population was pregnant women with high-risk pregnancies between June 2021 and November 2023. This study will be conducted in strict compliance with the STROBE statement and the Declaration of Helsinki, which was approved by the Ethics Committee (Approval n° 2022–596). In addition, all study participants had signed an informed consent form.

### Theoretical framework

The sample size needed for the study was calculated using G*Power software with α = 0.5, which showed that a minimum of 36 study subjects were needed in each group. The setting of calculation parameters: Effect size = 0.2, α err prob = 0.05, Power = 0.8, Ratio = 1:1. Study subjects were screened according to inclusion-exclusion criteria (inclusion criteria: The subjects were high-risk pregnant women (Age > 35 years or with gestational hypertension/diabetes mellitus) of 37‒42 weeks gestation who met the indications for planned induction of labor,^11^ with complete personal medical records such as pregnancy and prenatal examinations in the hospital, first and singleton pregnancies, and no contraindications to oxytocic drugs (specifically including ① malpositioned fetus. Central placenta praevia, partial placenta praevia, placental abruption. 3. Scarred uterus. Placental hypoplasia, intrauterine fetal distress. ⑤ Uncoordinated weak contractions and a spastic narrow ring of the uterus). Oxytocin (or combined with cervical balloon dilators) was used to induce labor. Exclusion criteria: Pregnant women with severe diseases such as coagulation dysfunction, liver and kidney metabolic disorders, vaginitis, autoimmune diseases, and systemic infectious diseases, were excluded).

### Participants

Ninety-three pregnant women with high-risk pregnancies from June 2021 to November 2023 were collected and analyzed retrospectively, of which 43 cases were induced by OXT (control group), and 50 cases were induced by OXT combined with cervical balloon dilators (observation group). The baseline data of two groups of pregnant women, shown in Table 1, revealed no significant inter-group differences (*p* > 0.05).

**Table 1:** There was no difference in the clinical baseline data between the two groups of pregnant women.

Groups	n	Age	Initial delivery (yes vs. no)	Week of pregnancy	Indications for induced labor				
					small amount of amniotic fluid	Hypertension in pregnancy	Gestational Diabetes Mellitus	Fetal growth restriction	Delayed pregnancy
Control group	43	29.49 ± 2.71	34 (79.1) vs. 9 (20.9)	39.86 ± 1.21	8 (18.6)	10 (23.3)	12 (27.9)	5 (11.6)	8 (18.6)
Observation group	50	28.90 ± 2.49	42 (84.0) vs. 8 (16.0)	39.42 ± 1.42	10 (20.0)	11 (22.0)	15 (30.0)	8 (16.0)	6 (12.0)
χ^2^/*t*		1.090	0.376	1.600	1.060				
p		0.279	0.539	0.113	0.901				

## Methods

Pregnant women in the control group received OXT to induce labor: 2.5 U of OXT was dissolved in 500 mL of % glucose injection for intravenous infusion, with an initial drip rate of 8 drops/min; the injection was adjusted every 15 minutes according to the frequency and intensity of uterine contractions, with the concentration controlled within 1 % and the drip rate within 40 drops/min until the duration of each contraction exceeded 30 s and there was one contraction every 5 minutes. Based on the above treatment, the observation group was further treated with cervical balloon dilators. After emptying the bladder and lying on the operating bed in the lithotomy position, the women was subjected to disinfection of the vulva and vagina and was guided to relax as much as possible. Then, a speculum was used to expose the cervix, and after disinfection, cervical balloon dilators were placed into the uterine cavity. The first balloon was completely inserted into the woman's uterine cavity, and the second balloon was placed in the cervical canal. Then, 80 milliliters of normal saline were injected into each of the two balloons, and the amount of fluid may be reduced as appropriate. The catheter was then gently pulled so that the balloon was held firmly against the cervix, and the balloon was removed when contractions and other mechanisms of delivery were initiated.

### Data collection and analysis

1) The Bishop scores (including evaluation items such as cervix opening, cervix position, cervical stiffness, effacement of the uterine cervix, presentation position, etc., with a total score of 13)^12^ of the mothers at the time of labor, before treatment, and 6 h after treatment were recorded; the score is directly proportional to the women's cervical ripening. 2) The first, second, and third stages of labor were counted, and the total duration of labor was calculated. 3) Systolic Blood Pressure (SBP), Diastolic Blood Pressure (DBP), and Heart Rate (HR) were recorded before and after treatment. 4) Enzyme-Linked Immunosorbent Assays (ELISAs) were performed before and after treatment to determine Norepinephrine (NE), Adrenaline (AD), Cortisol (Cor), Tumor Necrosis Factor-α (TNF-α), Interleukin-1β (IL-1β), IL-6, and Prostaglandin E2 (PGE2) levels. 5) The cesarean section rate and incidence of postpartum complications were analyzed.

### Statistical analysis

GraphPad 9.3 was employed for statistical analysis of data results. Qualitative and quantitative data were represented by [n (%)] and ($\bar{\chi }\pm s$), respectively; the comparison of the former used the χ^2^ test and that of the latter used the *t*-test. A value of *p* < 0.05 was considered statistically significant.

## Results

### The effect of labor induction was better in the observation group than in the control group

The labor time in the observation group was (15.06 ± 3.59)h, which was shorter compared with the control group (*p* < 0.05). The two groups were similar in the Bishop score (for the assessment of cervical ripening) before treatment (*p* > 0.05); but the score increased in both groups after treatment, especially in the observation group (*p* < 0.05, Table 2).

**Table 2:** The observation group had a shorter labor time and an elevated postpartum Bishop score.

Groups	n	Labor time (h)	Bishop score before treatment	Bishop score after treatment
Control group	43	18.49 ± 3.55	3.35 ± 0.75	5.93 ± 1.12^a^
Observation group	50	15.06 ± 3.59	3.52 ± 0.58	6.80 ± 1.12^a^
*t*		4.614	1.269	3.600
p		<0.001	0.208	<0.001

Note: ^a^ Indicates *p* < 0.05 compared to before treatment.

### Labor was shortened in the observation group

**Fig. 1:**
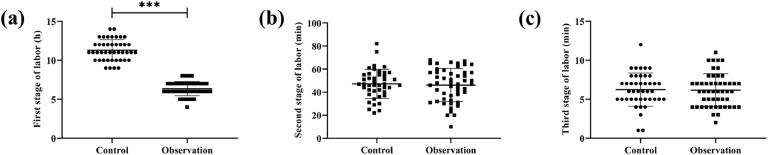
Comparison of labor times. (a) first stage of labor, (b) second stage of labor, (c) third stage of labor. *** p < 0.001.

According to statistics, there was no significant inter-group difference in the duration of the second and third stages of labor (*p* > 0.05); however, the duration of the first stage of labor was shorter in the observation group compared with the control group (*p* < 0.05, Fig. 1).

### The hemodynamic was more stable in the observation group

**Fig. 2:**
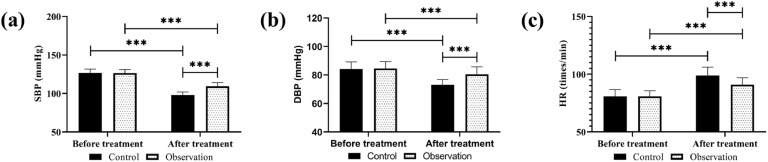
Comparison of hemodynamics. (a) SBP, (b) DBP, (c) HR. *** *p* < 0.001.

No significant inter-group differences were found in SBP, DBP, and HR before treatment (*p* > 0.05); SBP and DBP were reduced in both groups after treatment, with the SBP and DBP in the observation group being higher, while HR was elevated and lower in the observation group than in the control group (*p* < 0.05, Fig. 2).

### The stress response was less severe in the observation group than in the control group

**Fig. 3:**
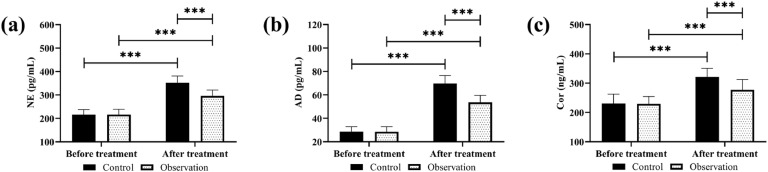
Comparison of stress responses. (a) NE, (b) AD, (c) Cor. *** *p* < 0.001.

The two groups showed no marked differences in pre-treatment levels of stress response markers (*p* > 0.05); a significant increase in NE, AD, and Cor was observed in both groups after treatment, with lower levels of these stress response indexes in the observation group compared with the control group (*p* < 0.05, Fig. 3).

### The observation group showed milder inflammation than the control group

**Fig. 4:**
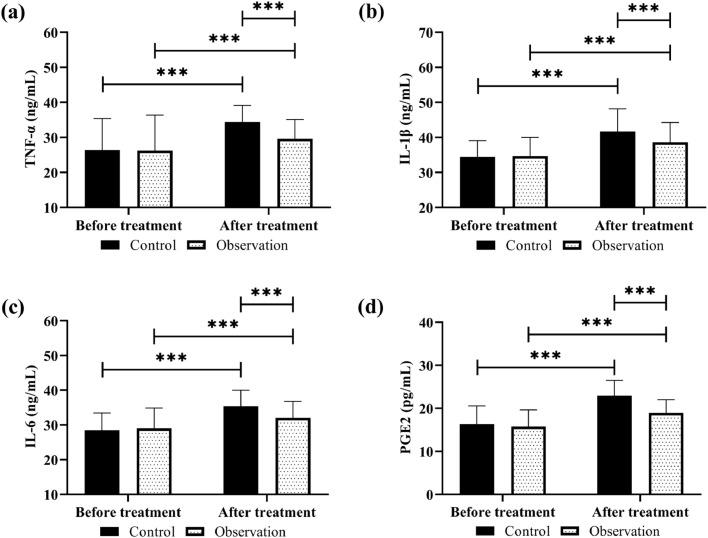
Comparison of inflammatory responses. (a) TNF-α, (b) IL-1β, (c) IL-6, (d) PGE2. ***p < 0.001.

The detection of inflammatory factors revealed no significant inter-group difference in TNF-α, IL-1β, IL-6, and PGE2 before treatment (*p* > 0.05); after treatment, TNF-α, IL-1β, IL-6, and PGE2 all increased in both groups, but with lower levels in the observation group compared to the control group (*p* < 0.05, Fig. 4).

### The delivery safety was higher in the observation group versus the control group

The cesarean section rate in the observation group was 41.9 %, which was reduced compared to the control group (*p* < 0.05); in addition, the incidence of postpartum complications in the observation group was 18.0 %, which was not significantly different from that in the control group (*p* > 0.05, Table 3).

**Table 3:** Comparison of safety.

Groups	n	Vaginal delivery	Cesarean section	Cervical laceration	Postpartum fever	Postpartum hemorrhage	Vaginal hematoma	Pelvic infection	Overall incidence
Control group	43	39 (78.0)	11 (54.1)	2 (4.0)	2 (4.0)	3 (6.0)	1 (2.0)	1 (2.0)	9 (18.0)
Observation group	50	25 (22.0)	18 (41.9)	3 (7.0)	2 (4.7)	3 (7.0)	2 (4.7)	3 (7.0)	11 (25.6)
χ^2^		4.249							0.787
p		0.039							0.375

## Discussion

For high-risk pregnant women affected by underlying diseases, the degree of postpartum stress damage and inflammation is the key to determining their postpartum health. In this study, OXT combined with cervical balloon dilators effectively alleviated postpartum stress injury and inflammatory reaction in high-risk pregnant women, which undoubtedly provides a more reliable safety guarantee for future treatment of high-risk pregnancies.

First, by analyzing the labor process, it was found that the observation group had shortened labor time and improved cervical ripening, indicating that the combination of OXT and cervical balloon dilators can provide a more reliable guarantee for labor induction. Similarly, the duration of the first stage of labor and the total duration of labor were shorter in the observation group compared with the control group, confirming that OXT in combination with cervical balloon dilators has a more significant effect on labor induction. Previous studies have repeatedly validated the positive effect of OXT combined with cervical balloon dilators on labor induction,^13^^,^^14^ which can support the results of this paper. The reason is that the cervical balloon dilators can shorten the labor time by grasping the timing of amniotic sac rupture and releasing amniotic fluid by artificially rupturing the amniotic sac, plus the contractile effect of OXT to promote the labor process, which is conducive to accelerating the cervical ripening of the mother, contributing to better overall labor induction efficacy. In the comparison of hemodynamics, SBP and DBP in the observation group were higher, while HR was lower, suggesting more stable hemodynamics in the observation group. As the authors all know, the control of adverse stress during labor induction is also an important part of safety assessment, and hemodynamic parameters are effective indicators for this evaluation.^15^ Of them, the control and reduction of the fluctuation range of SBP, DBP, and HR is the necessary basis and premise, which is the most intuitive reflection of the safety of the labor induction process.^16^ This may be because more attention has been paid to the natural delivery mechanism of pregnant women during the promotion of cervical ripening and induction of labor by OXT plus cervical balloon dilators. In addition, cervical balloon dilators are safer more natural and progressive in promoting cervical ripening and induction of labor, thus having a better impact on the hemodynamics of pregnant women. In Huang K et al. study, they also mentioned that cervical balloon dilators have an excellent hemodynamic protective effect on pregnant women with hypertensive disorders,^17^ which can also support the results of the current study.

On the other hand, in the comparison of stress indexes and inflammatory factors between the two groups, NE, AD, Cor, TNF-α, IL-1β, IL-6, and PGE2 were all found to be lower in the observation group compared with the control group, which indicates that OXT combined with cervical balloon dilators also has a more significant effect in relieving stress injury and inflammatory reaction in high-risk pregnant women. The authors believe that balloon compression can compress the muscle fiber vessels of the uterine muscle wall and promote the closure of blood sinuses for hemostasis, and its combined use with OXT can play a synergistic role, thus ameliorating women' abnormal coagulation function and reducing the risk of postpartum hemorrhage.^18^ Postpartum hemorrhage, however, is a traumatic stress response that stimulates the excitation of the body's sympathetic nervous system and promotes increased synthesis of endocrine hormones such as NE and AD.^19^ Therefore, OXT combined with cervical balloon dilators can reduce the body's stress response by lowering the possibility of postpartum hemorrhage in pregnant women, thereby promoting a decrease in stress-related hormone synthesis. Moreover, OXT combined with cervical balloon dilators can change uterine pressure, promote fetal delivery with amniotic fluid, avoid the negative effects such as fatigue caused by prolonged labor on the mother, and reduce the damage caused by excessive cervical contraction to the myometrium and uterus,^20^ which also greatly avoids the possibility of the release of inflammatory mediators from organs and tissues due to stress reaction. Therefore, the levels of inflammatory factors in the observation group were also significantly lower than those in the control group. In a study by Hubbell RD on the treatment of patients with eustachian tube dysfunction, their findings showed that balloon dilators had less stress damage and less discomfort for patients.^21^ Meanwhile, the findings of Poe D et al. confirm the lower inflammatory response after pharyngeal balloon dilatation using balloon dilators,^22^ which is in line with our view.

Finally, the decrease in cesarean section rate among pregnant women in the observation group further confirms that the combination of OXT and cervical balloon dilators can help shorten cervical ripening time and labor duration and improve the natural delivery rate. In the study of Jamaluddin A et al., they used cervical balloon dilators during planned delivery in full-term mothers and found that their effect of promoting cervical ripening was more significant than that of OXT alone, which could increase the rate of vaginal delivery and help shorten the duration of labor.^23^ However, there was no statistical inter-group difference in postpartum complications, which may be due to the use of cervical balloon dilators, a non-drug intervention that mainly promoted cervical ripening and dilation by mechanical action, without adverse drug reactions.

Due to limited conditions, this study still shows many limitations. For example, the absence of neonatal condition investigation and the short follow-up period of pregnant women make it difficult to assess the long-term effects of OXT in combination with cervical balloon dilators. In future research, the authors should also include more objective indicators, such as coagulation function and sex hormones, to obtain more comprehensive results for clinical reference.

## Conclusion

OXT combined with cervical balloon dilators can effectively shorten the labor process of pregnant women with high-risk pregnancies, improve the efficiency of labor induction, and relieve postpartum stress injury and inflammatory responses, with high clinical application value, which is recommended to be widely used in future labor induction in high-risk pregnancies.

## Ethical approval

The study protocol was approved by the Ethics Committee of Hainan Affiliated Hospital of Hainan Medical University (Approval No:2022–596).

## Availability of data and materials

The data that support the findings of this study are available from the corresponding author upon reasonable request.

## Authors’ contributions

HM.G. conceived and designed the study, RH.L. and MD.X. wrote and revised the manuscript, YH.Y. and XQ.H. collected and analyzed data, RH.L. and MD.X. made equal contributions in this work as co-first authors. All authors read and approved the final submitted manuscript.

## Funding

Not applicable.

## Conflicts of interest

The authors declare no conflicts of interest.
